# ﻿*Xanthophytumantoanense* (Rubiaceae, Ophiorrhizeae), a new species from Vietnam

**DOI:** 10.3897/phytokeys.250.137482

**Published:** 2024-12-30

**Authors:** Hieu Cuong Nguyen, Xuan Bach Nguyen-Le, Quoc Dat Nguyen, Tran Quoc Trung Nguyen, Hong Truong Luu

**Affiliations:** 1 Southern Institute of Ecology, Institute of Applied Materials Science, Vietnam Academy of Science and Technology, Ho Chi Minh City, Vietnam Institute of Applied Materials Science, Vietnam Academy of Science and Technology Ho Chi Minh Vietnam; 2 Graduate University of Science and Technology, Vietnam Academy of Science and Technology, Hanoi, Vietnam Graduate University of Science and Technology, Vietnam Academy of Science and Technology Hanoi Vietnam

**Keywords:** An Toan Nature Reserve, Central Vietnam, endemic, head-like inflorescence, indumentum, setose

## Abstract

*Xanthophytumantoanense* is described as a new species endemic to Central Vietnam. It is morphologically closest to *X.capitatum* in having setose hairs on the abaxial leaf surface and a pedunculate head-like inflorescence but differs from the latter by a number of characteristics: shorter stem, 3-lobed stipules, narrowly lanceolate leaf blades with a cuneate-oblique base and 20–22 pairs of secondary veins, 3.5–4.5 cm long peduncle, spatulate calyx lobes, larger corollas with a 5–5.4 mm long tube and 2.4–3 mm long lobes, and an apically hairy style. A detailed description, an illustration, and information on distribution, ecology and phenology, and a provisional assessment of the conservation status of the new species are provided.

## ﻿Introduction

*Xanthophytum* was initially proposed as a genus of the Rubiaceae by Blume (1823) but was only validly described in his subsequent work (Blume 1826). Its taxonomic position varied according to authors, being assigned to different tribes over time, such as Pomazoteae (Bremekamp 1952), Hedyotideae (Robbrecht 1988), and either Hedyotideae or Ophiorrhizeae (Tange 1995, published 1996). More recently, it has been placed in Ophiorrhizeae (Bremer and Manen 2000). Most recent molecular phylogenetic analyses confirm its placement in Ophiorrhizeae (Razafimandimbison and Rydin 2019, 2024). The genus was revised by Axelius (1990), who recognized 30 species. Later, Tange (1995 [published 1996]) asserted that *Myrioneuronborneense* Stapf (Stapf 1894) and *Siderobombyxkinabaluensis* Bremek. (Bremekamp 1947) were synonymous and should be included in *Xanthophytum*, based on their distinctive small brown seeds with tuberculate exotesta cell thickenings and ferruginous hairs. Consequently, he reduced the monospecific genus *Siderobombyx* to a synonym of *Xanthophytum*. Since the combination *X.borneense* (Valeton) Axelius was already established (Axelius 1990), he provided the new combination *X.kinabaluense* (Stapf) Tange for this species. However, the correct name should be *X.kinabaluense* (Bremek.) Tange, as the epithet was originally used by Bremekamp (1947). Additionally, he described a new species, *X.bullatum* Tange, from Sarawak, Malaysia. Currently, the genus comprises 32 accepted species distributed across tropical and subtropical Asia to the Southwest Pacific (POWO 2024).

Prior to this study, five species of *Xanthophytum* had been documented in Vietnam (Axelius 1990; Pham-Hoang 2003; Chen and Taylor 2011): *X.attopevense* (Pierre ex Pitard) H.S.Lo (Lo 1986; Pitard 1922), *X.balansae* (Pitard) H.S.Lo (Lo 1986; Pitard 1922), *X.johannis-winkleri* Merrill (Merrill 1937), *X.kwangtungense* (Chun & F.C.How) H.S.Lo (Chun and How 1939; Lo 1986), and *X.polyanthum* Pitard (Pitard 1922). Notably, the distribution of *X.attopevense* and *X.johannis-winkleri* in Vietnam remains uncertain. The collection *d’Alleizette s.n.*, June 1909 (P) from Nha Trang, was provisionally assigned to *X.attopevense* and may represent an unknown species (Axelius 1990). Meanwhile, *X.johannis-winkleri* was reported by Pham-Hoang (2003) to occur in Nha Trang, but no vouchered specimens were included; this record is likely followed by POWO (2024). Axelius (1990) documented this species’ distribution in West Borneo, which is considerably disjunct from mainland Southeast Asia. Given this context, these two species should be excluded from the flora of Vietnam until their distribution in the country is reliably confirmed.

During our botanical inventory at An Toan Nature Reserve in Central Vietnam in May 2023, we encountered a *Xanthophytum* plant exhibiting a striking red ferruginous indumentum on its stem, leaves, and head-like inflorescence. Detailed morphological examination confirmed that it represents a new species, which we describe below.

## ﻿Materials and methods

The studied material was collected from Binh Dinh Province, Central Vietnam. Specimens were sampled and processed using methods described by the Royal Botanic Gardens, Kew (Bridson and Forman 1999). Herbarium acronyms follow Thiers (2024). Detailed photographs and the description of taxonomically important characters of the new species were based on fresh material. Taxonomic identification was based on vegetative and reproductive morphological characters following the aforementioned literature, primarily Axelius (1990) and Chen and Taylor (2011).

## ﻿Taxonomic treatment

### ﻿ Xanthophytum antoanense

Taxon classificationPlantaeGentianalesRubiaceae

Luu, H.C.Nguyen, X.B.Nguyen-Le & Q.D.Nguyen sp. nov.

E01F4A57-422E-56A2-A730-0172AC5BC43C

urn:lsid:ipni.org:names:77354340-1

Fig. 1

#### Type.

Vietnam • Binh Dinh Province, An Lao District, An Toan Commune, An Toan Nature Reserve, coordinates 14°31'31"N, 108°42'48"E, 838 m elevation, 17 May 2023, Nguyen Le Xuan Bach, Nguyen Quoc Dat, Nguyen Hieu Cuong *BN1015* (Holotype: SGN!, barcode SGN006396).

**Figure 1.**
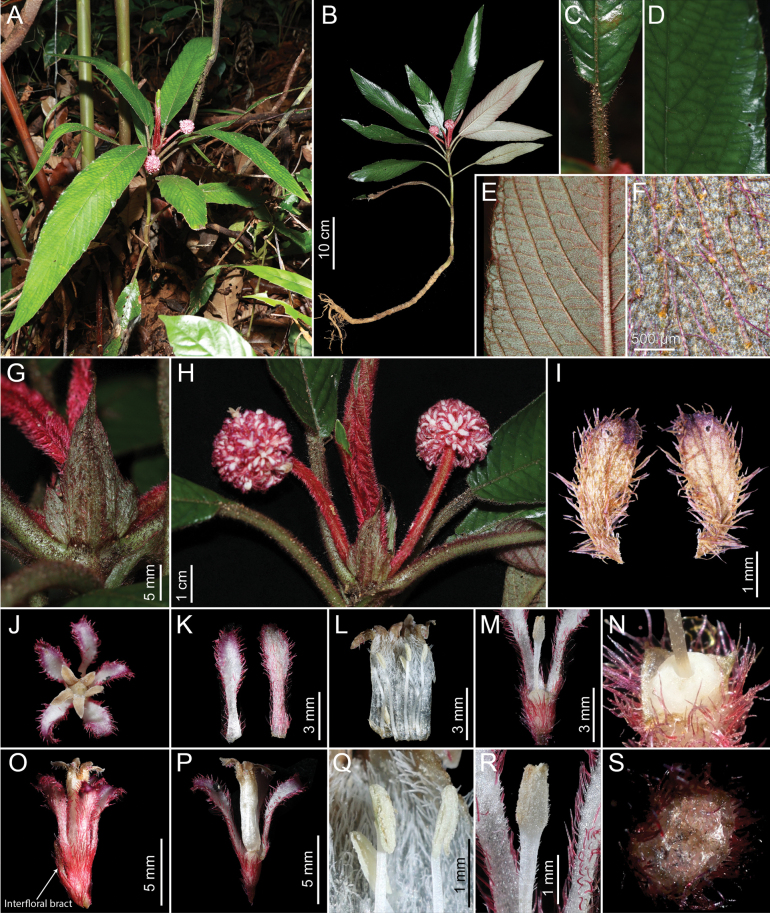
*Xanthophytumantoanense***A** plant in situ **B** whole plant **C** leaf base **D** leaf, adaxial surface **E** leaf, abaxial surface **F** setose hairs on abaxial leaf surface (dried) **G** stipule **H** inflorescences **I** bracts (dried) **J** flower, top view **K** calyx lobes **L** opened corolla showing stamens **M** pistil **N** ovary, disc and base of style **O** flower, side view, with an interfloral bract (arrow) **P** corolla and calyx, some calyx lobes removed **Q** anthers **R** stigma (corolla removed) **S** cross-section of ovary.

#### Diagnosis.

The new species is morphologically closest to *X.capitatum* Valeton but differs from the latter by its 3-lobed (vs. unlobed) stipules, narrowly lanceolate (vs. oblong to obovate) leaf blades with a cuneate-oblique (vs. symmetrically cuneate to attenuate) base and 20–22 (vs.11–16) pairs of secondary veins, 3.5–4.5 (vs. 0.5–3) cm long peduncle, spatulate (vs. bluntly triangular) calyx lobes, larger flowers with a 5–5.4 (vs. c. 1.8) mm long corolla tube and 2.4–3 (vs. c. 0.7) mm long corolla lobes, and an apically hairy (vs. glabrous) style.

#### Description.

Monocaul dwarf shrublet, sub-herbaceous, to 50 cm tall. ***Stems*** densely hairy, hairs septate, red, c. 1 mm long. ***Leaves*** simple, opposite, decussate; ***petiole*** 4–6 cm long, c. 0.35 cm in diameter, densely hairy with hairs septate, red, c. 1.5 mm long; ***lamina*** elongate-lanceolate, 13–24 cm long, 3–4.5 cm wide, adaxially green, glabrous, abaxially gray, densely hairy, hairs setose, red, c. 1.5 mm long; base cuneate-oblique, apex acute; ***venation*** pinnate, midrib prominent on both surfaces, adaxially sparsely and abaxially densely hairy, hairs c. 1.5 mm long, secondary veins 20–22 pairs, weakly brochidodromous, tertiary veins abaxially prominent. ***Stipules*** 1.7–2.5 cm long, 1.2 cm at widest, ovate, acuminate, slightly 3-lobed, abaxially densely hairy, hairs septate, red, c. 1.5 mm long. ***Inflorescences*** axillary, pseudo-terminal heads, erect then nodding, heads c. 2 cm in diameter, many-flowered; ***peduncles*** 3.5–4.5 cm long, 0.2–0.3 cm in diameter, densely hairy, hairs septate, red, c. 1.3 mm long. ***Bracts*** oblanceolate, 3.0–3.2 mm long, 1.1–1.3 mm wide, white, apically red-violet, ciliate, abaxially densely hairy, hairs septate, red, c. 1 mm long. ***Interfloral bracts*** linear, c. 3.5 mm long, 0.25 mm wide, white, abaxially densely hairy, hairs septate, red, c. 0.6–0.8 mm long. ***Flowers*** bisexual, probably heterostylous, sessile, c. 1.1 cm long. ***Calyx*** 5-lobed, lobes spatulate, white, apically red, 7–8 mm long, c. 1.6 mm at widest, adaxially glabrous, abaxially densely hairy, hairs septate, red, c. 1 mm long. ***Corolla*** white, turning brown, tubiform with spreading lobes; ***tube*** 5–5.4 mm long, 1.2–1.4 mm in diameter, outside glabrous, inside densely white tomentose on upper ¾, hairs 0.4–0.5 mm long; ***lobes*** 5, 2.4–3 mm long, 0.7–1 mm at widest, oblong-lanceolate, acute, outside white to red setose with 0.5–0.6 mm long stiff hairs, inside papillose. ***Stamens*** 5, inserted at corolla base, 5.3–5.5 mm long; filaments c. 5 mm long, glabrous; anthers c. 1 mm long, dorsifixed, longitudinally dehiscent. ***Pistil*** 4.8–5.0 mm long; ***disc*** white, hemispherical, c. 0.5 mm high; ***ovary*** 2-celled, c. 2 mm high, 1.5 mm wide, densely red septate hairy; ***ovules*** numerous on a globose, axile placenta in each cell; ***style*** 2.7–2.8 mm long, gradually thickened towards the apex, sparsely hairy on upper half; ***stigma*** 2-lobed, lobes lanceolate, with obtuse apex, c. 1.3–1.4 mm long. ***Fruit*** unknown.

#### Etymology.

The species epithet is derived from the location where the new taxon was discovered.

#### Vernacular name.

*Hoàng cành An Toàn* (Vietnamese).

#### Phenology.

Flowering observed in May; fruiting is unknown.

#### Distribution and ecology.

The species is known only from An Toan Nature Reserve (Binh Dinh Province, Vietnam). It grows on moist fertile soils in lower montane evergreen forests at elevations around 850 m. The forest is dominated by Rubiaceae, Fagaceae, Lauraceae, Myrtaceae, Clusiaceae, and Fabaceae.

#### Preliminary IUCN conservation status.

Data Deficient (IUCN Standards and Petitions Subcommittee 2024). The new species appears to be rare and was discovered in a small population. Its actual distribution and population size cannot be assessed at present, but the species is likely to occur in adjacent forests in Gia Lai and Quang Ngai Provinces. Further surveys are necessary to determine its precise conservation status.

#### Taxonomic notes.

Axelius (1990) identified ferruginous indumentum as a synapomorphy for *Xanthophytum* species. Despite variations in color and density among species, the indumentum generally consists of two kinds of hairs: multicellular (septate) and unicellular. Septate hairs are found on vegetative parts and the exterior of the calyx and corolla, while unicellular hairs are located inside the corolla. These hairs are further classified into four types, one of which is setose hairs that are characterized as stiff, ferruginous, approximately 0.5 mm long, entirely septate with thick, smooth cell walls. Confined to vegetative parts, setose hairs have an upper part filled with a reddish substance and a swollen basal cell that protrudes from the leaf surface. To date, only two *Xanthophytum* species, *X.capitatum* and *X.setosum* Axelius (Axelius 1990), have been reported to possess setose hairs on their leaves. Therefore, our new taxon represents the third species in this group. Its setose hairs are up to 1.5 mm long, three times as long as those in the other species.

In *Xanthophytum*, flower morphology varies across species, some species having heterostylous flowers and others having homostylous flowers, with different placements of stamens and stigma, as well as varying hair-ring widths inside the corolla. However, many species remain undocumented due to lack of flowering material (Axelius 1990; Chen and Taylor 2011). According to Axelius (1990), in homostylous species with narrow hair-rings, anthers are positioned at the level of the ring with the stigma positioned just above the ring; for those with broad hair-rings, anthers are positioned at the ring’s upper margin with the stigma clearly above the hair-ring. Most heterostylous species with narrow hair-rings have the anthers positioned at the level of the ring and the stigma positioned clearly above it, while species with broad hair-rings show more variation: the stigma can be positioned above or at the level of the ring, and anthers in brevistylous flowers may be above, at, or below the hair-ring, whereas in longistylous flowers, anthers are usually at the level of the ring. Our specimen, with a broad hair-ring, has the anthers at the upper part and the stigma at the lower part of the ring, similar to brevistylous flowers in other species like *X.cylindricum* Axelius (Axelius 1990). We thus assume our species is heterostylous, and our studied specimen is likely brevistylous, as we have not encountered longistylous flowers.

*Xanthophytumantoanense* is morphologically closest to *X.capitatum* (Valeton 1910), sharing the presence of setose hairs on the abaxial leaf surface and a pedunculate head-like inflorescence. However, the two species can be distinguished by key morphological characteristics as outlined in the diagnosis. Additionally, *X.antoanense* differs in having ovate, slightly 3-lobed and 1.7–2.5 cm long stipules (vs. triangular to ovate and c. 0.7 cm long in *X.capitatum*), 4–6 (vs. c. 3.5) cm long petioles, larger flower heads (c. 2 vs. c. 1.5 cm in diameter), adaxially glabrous and 7–8 mm long calyx lobes (vs. pubescent and c. 0.6 mm long), oblong-lanceolate (vs. triangular) corolla lobes, and brevistylous flowers with 5.3–5.5 (vs. c. 2) mm long stamens and a 2.7–2.8 (vs. c. 1.4) mm long style. Furthermore, the corolla tube of brevistylous flowers in *X.antoanense* is densely hairy on the upper three-quarters of the inside, whereas in *X.capitatum*, it has a c. 0.6 mm high ring of hairs in the upper part of the tube.

*Xanthophytumsetosum* is easily distinguishable from the new taxon as it has a paniculate inflorescence. It further differs from the new species in having smaller leaf blades (7–16 cm long in *X.setosum* vs. 13–24 cm long) with 12–19 (vs. 20–22) pairs of secondary veins, shorter petioles (≤ 4 vs. 4–6) cm long, c. 1 cm long and unlobed (vs. 1.7–2.5 cm long and 3-lobed) stipules, minute (vs. c. 3.5 mm long) bracts, an adaxially densely pubescent (vs. glabrous) calyx with bluntly triangular and c. 1 mm long (vs. spatulate and 7–8 mm long) lobes, smaller brevistylous flowers (longistylous flowers unknown) with c. 1.8 (vs. 5–5.4) mm long corolla tube and c. 1.2 (vs. 2.4–3) mm long corolla lobes, shorter stamens (c. 2.8 vs. 5.3–5.5 mm long), and a c. 1.2 mm long and glabrous (vs. 2.7–2.8 mm long and apically hairy) style. Finally, the corolla tube in *X.antoanense* is densely hairy on the upper three-quarters of the inside and glabrous on the outside, whereas in *X.setosum*, it has a narrow ring of hairs below the throat on the inside and is sparsely hairy on the outside.

The leaf size and shape, head-like pedunculate inflorescence, and spatulate calyx lobes in *Xanthophytumantoanense* are reminiscent of those in *X.attopevense* from Laos. However, *X.attopevense* differs in several key characteristics: it has a cuneate to attenuate (vs. cuneate-oblique in *X.antoanense*) leaf base, shorter petioles (c. 2 vs. 4–6 cm long), c. 1.5 cm long and unlobed stipules (vs. 1.7–2.5 cm long and 3-lobed), flower heads c. 1.5 (vs. c. 2) cm in diameter, up to 1.5 (vs. 3.5–4.5) cm long peduncles, adaxially hairy and 1.5–2.5 mm long (vs. glabrous and 7–8 mm long) calyx lobes, a c. 2 (vs. 5–5.4) mm long corolla tube, and c. 1 (vs. 2.4–3) mm long corolla lobes. Notably, *X.attopevense* was not reported to have setose hairs on its leaf blades, although this type of hair is depicted in a drawing illustrating fruits attached to the type specimens *Harmand 1099*, collected in February 1877, Attopeu, Bassac, Laos (P, barcodes 02436251 and 02436253) (JSTOR 2024).

The morphological differences among these four species are summarized in Table 1.

**Table 1.** Key morphological differences between *Xanthophytumantoanense* and its closest congeners (based on Axelius 1990; Chen and Taylor 2011).

Characteristics	*X.antoanense*	*X.attopevense*	*X.capitatum*	*X.setosum*
Indumentum on stem	septate	sericeous to pilose	septate and setose	septate and setose
Stipules	ovate, 1.7–2.5 cm long, 3-lobed, hairs septate	ovate, c. 1.5 by 0.8 cm, unlobed, hairs sericeous or pilose to glabrescent	triangular to ovate, 0.7 cm long, unlobed, hairs septate	lanceolate, triangular or ovate, c. 1 cm long, unlobed, hairs septate and setose
Leaf blade	13–24 × 3–4.5 cm, narrowly lanceolate, abaxially with dense red setose hairs, base cuneate-oblique	11–20 × 3–5 cm, broadly lanceolate to oblong, abaxially with sericeous hairs, base cuneate to attenuate	8–18.5 × 2.5–7 cm, oblong to obovate, abaxially with setose hairs, base cuneate to attenuate	7–16 × 2.5–6.6 cm, lanceolate to oblong, abaxially with setose hairs, base cuneate to attenuate
Leaf secondary veins	20–22 pairs	20–24 pairs	11–16 pairs	12–19 pairs
Petioles	4–6 cm long, hairs septate	2 cm long, hairs sericeous	3.5 cm long, hairs setose	≤4 cm long, hairs setose
Inflorescence	head-like, heads 2 cm in diameter	head-like, heads c. 1 cm in diameter	head-like, heads ≤1.5 cm in diameter	paniculate
Peduncles	3.5–4.5 cm long, hairs septate	≤1.5 cm long, hairs sericeous	0.5–3 cm long, hairs setose	≤3.5 cm long, hairs septate and setose
Bracts	oblanceolate, 3.0–3.2 mm long	triangular, 1.5–3 mm long	narrow, ≤1 cm long	minute
Calyx	adaxially glabrous	adaxially glabrous	adaxially densely pubescent	adaxially densely pubescent
Calyx lobes	spatulate, 7–8 mm long	band-shaped to broadly spatulate, 1.5–2.5 mm long	bluntly triangular, c. 0.6 mm long	bluntly triangular, c. 1 mm
Corolla tube of brevistylous flowers	5–5.4 mm long, inside densely hairy on upper ¾, outside glabrous	c. 2 mm long, inside with a ring of hairs at the upper part, outside glabrous	1.8 mm long, inside with a 0.6 mm high ring of hairs in the upper part of the tube, outside glabrous	c. 1.8 mm, inside with a narrow ring of hairs a bit down from the throat, outside sparsely hairy
Corolla lobes	oblong-lanceolate, 2.4–3 mm long	shape unknown, c. 1 mm long	triangular, 0.7 mm long	triangular, 1.2 mm long
Stamens of brevistylous flowers	5.3–5.5 mm long	c. 2 mm long	c. 2 mm long	c. 2.8 mm long
Style of brevistylous flowers	2.7–2.8 mm long, sparsely hairy at upper half	c. 2.5 mm long, glabrous	c. 1.4 mm long, glabrous	c. 1.2 mm long, glabrous

### ﻿Key to the 4 presently known Vietnamese species of *Xanthophytum*

**Table:** 

1	Inflorescence head-like	**2**
–	Inflorescence paniculate	**3**
2	Lateral veins 9–16 pairs	***X.kwangtungense***
–	Lateral veins ≥ 20 pairs	***X.antoanense***
3	Petioles up to 1 cm long; lateral veins 11–15 pairs	***X.balansae***
–	Petioles up to 5 cm long; lateral veins 14–25 pairs	***X.polyanthum***

## Supplementary Material

XML Treatment for Xanthophytum antoanense

## ﻿Additional information

### Author ORCIDs

Hieu Cuong Nguyen https://orcid.org/0000-0001-8707-2944

Xuan Bach Nguyen-Le https://orcid.org/0000-0001-8391-338X

Quoc Dat Nguyen https://orcid.org/0000-0002-3687-1567

Tran Quoc Trung Nguyen https://orcid.org/0000-0003-4366-2381

Hong Truong Luu https://orcid.org/0000-0002-7036-7081

### Data availability

All of the data that support the findings of this study are available in the main text.
